# Radiotherapy for Ductal Carcinoma In Situ: Toxicity, Quality of Life, and Decisional Regret at a Tertiary Cancer Center

**DOI:** 10.3390/cancers18121946

**Published:** 2026-06-16

**Authors:** Husam Abdulsattar Alhasan, Markus Anton Schirmer, Leif Hendrik Dröge, Carla Marie Zwerenz, Manuel Guhlich, Sandra Donath, Stefan Rieken, Gunther Felmerer, Stephanie Bendrich

**Affiliations:** 1 Department of Radiation Oncology, University Medical Center Goettingen (UMG), Robert-Koch-Str. 40, 37075 Goettingen, Germany; h.alhasan@stud.uni-goettingen.de (H.A.A.); markus.schirmer@med.uni-goettingen.de (M.A.S.); hendrik.droege@med.uni-goettingen.de (L.H.D.); carlamarie.zwerenz@med.uni-goettingen.de (C.M.Z.); manuel.guhlich@med.uni-goettingen.de (M.G.); sandra.donath@med.uni-goettingen.de (S.D.); stefan.rieken@med.uni-goettingen.de (S.R.); 2 Comprehensive Cancer Center Göttingen (G-CCC), University Medical Center Goettingen (UMG), Robert-Koch-Str. 40, 37075 Goettingen, Germany; gunther.felmerer@med.uni-goettingen.de; 3 Department for Trauma Surgery, Orthopedics and Plastic Surgery, Division of Plastic Surgery, University Medical Center Goettingen (UMG), Robert-Koch-Str. 40, 37075 Goettingen, Germany

**Keywords:** ductal carcinoma in situ, adjuvant radiotherapy, long term toxicity, quality of life, decisional regret, recurrence, risk factors

## Abstract

**Purpose:** Adjuvant radiotherapy (RT) for ductal carcinoma in situ (DCIS) remains a widely discussed topic. While it has been shown to significantly reduce recurrence rates, it does not improve overall survival (OS) and may therefore lead to overtreatment in some cases. This study aimed to assess patient acceptance, quality of life (QoL), and decisional regret (DR) in relation to late adverse effects. **Methods**: Clinical data from patients who underwent RT for DCIS between 2008 and 2020 were retrospectively collected, while follow-up questionnaires evaluated QoL and DR. High-risk DCIS was defined as tumor size ≥ 2.5 cm, poor differentiation (G3), age ≤ 50, and symptomatic diagnosis. Low-risk disease was defined as tumor size < 2 cm, G1/G2, age > 50, and mammographic diagnosis. Patients who did not meet all criteria for either category were classified as intermediate risk, therefore reflecting everyday work and clinical practice. Questionnaire data were analyzed using both scale-level and item-level approaches and Ipsilateral breast recurrence-free survival (IBRFS) rates were calculated using the Kaplan–Meier method. **Results**: Among 307 cases offered RT, only 24 declined, indicating high treatment acceptance; an additional 20 cases lacked sufficient documentation, did not receive RT at our center despite acceptance, or discontinued RT prematurely. During the study period, late toxicities occurred at acceptable frequencies: fibrosis in 6.1%, hyperpigmentation in 6.8%, telangiectasia in 8%, and edema in 1.5% of cases with excellent control rates of 90.0%. The response rate to the EORTC QLQ-C30 and Ottawa Decisional Regret Scale (DRS) was 35.4%, of whom 70 cases belonged to the intermediate-risk group. These respondents reported high QoL and low levels of DR. **Conclusions**: RT for intermediate grade DCIS is accepted and tolerated well, and neither late adverse effects nor QoL outcomes were associated with DR. These findings support recommending RT in this borderline-indication group.

## Simple Summary

DCIS is the sole precancerous condition subjected to treatment as aggressively as invasive carcinoma, which poses a danger of overtreatment for a subset of individuals. The cohort of patients with intermediate-risk DCIS is particularly noteworthy, as the determination for treatment de-escalation is more challenging in this population. Consequently, this study aimed to evaluate patient acceptance, quality of life, and decision regret concerning late consequences. Our findings suggest that, in general, radiotherapy is widely accepted in this group, patients indicate satisfactory quality of life, and there is no correlation between treatment regret and the onset of late sequelae, combined with excellent control rates.

## 1. Introduction

Ductal carcinoma in situ (DCIS) is a non-invasive form of breast cancer in which malignant cells within the mammary ducts have not penetrated the basement membrane [1]. Over the past decades, the incidence of DCIS has risen markedly, largely due to widespread implementation of mammography screening. In many Western countries, DCIS now accounts for approximately 20–25% of all newly diagnosed breast cancers. Each year, around 6500 women in Germany and about 56,000 women in the United States are diagnosed with DCIS [2,3]. DCIS itself is generally considered non-life-threatening; however, a subset of cases progresses to invasive disease, contributing to a breast-cancer-specific mortality of around 3% at 20 years [4]. Fatal outcomes may arise from invasive progression that is either detected as an ipsilateral recurrence or remains clinically unrecognized until metastatic disease develops. This creates a clinical dilemma: balancing the risk of overtreatment of indolent lesions against the need to prevent invasive progression [5]. To address this, we conducted an analysis to identify patient-, tumor-, and treatment-related factors associated with an increased risk of recurrence.

The primary treatment of DCIS is surgical, typically breast-conserving surgery (BCS) or, in cases of extensive disease, mastectomy. When surgical margins are close or positive, re-excision is usually indicated to ensure adequate local control [6]. Adjuvant radiotherapy (RT), delivered as normo- or moderately hypo-fractionated RT after BCS, reduces the relative local recurrence risk by around 50% over 10 years but has not been shown to improve overall survival (OS). A key focus of present research is defining patient groups for whom RT might be safely omitted, in order to avoid unnecessary side-effects and reduce overtreatment and resource use in low-risk disease [7,8]. Other management strategies include molecular profiling, endocrine therapy, hypo-fractionated RT and, in selected cases, active surveillance as a potential alternative to surgery [9]. This highlights the need to optimize treatment strategies for DCIS, especially considering the burden associated with a minimum of 3 and up to 6 weeks of RT visits and related costs, acute side effects such as breast pain and fatigue, and potential long-term toxicities including breast fibrosis, cardiac disease, and secondary cancers [10].

The ongoing uncertainty regarding which patients truly benefit from specific treatments underscores the importance of shared decision-making (SDM) and clear communication of risks and benefits to patients with DCIS. Therefore, we additionally performed an evaluation of quality of life (QoL) and decisional regret (DR). These aspects, particularly regarding RT for DCIS, remain underrepresented in the current literature and therefore represent the main focus of our study.

## 2. Patients and Methods

### 2.1. Patient Selection

**Figure 1:**
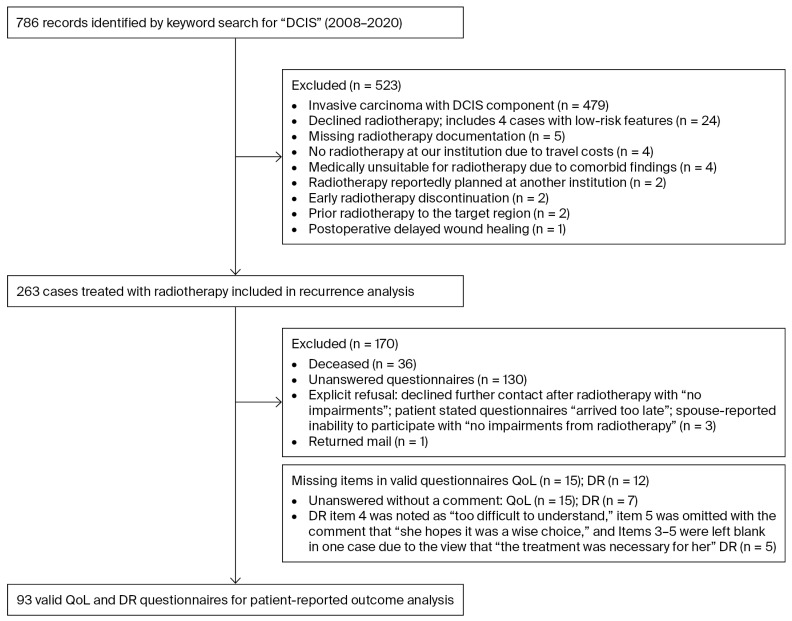
Patient selection flowchart. Records identified by DCIS keyword screening (2008–2020) with subsequent inclusion for recurrence analysis and patient-reported outcomes.

In this monocentric study, clinical data of patients with DCIS treated at the Department of Radiation Oncology University Medical Center Göttingen, Germany, between 2008 and 2020 were analyzed retrospectively. Patients were initially identified by a systematic keyword search for “DCIS”. This resulted in a cohort of 786 cases, corresponding to patients with invasive breast cancer with DCIS components as well as pure DCIS. Since some patients had bilateral disease, the number of cases slightly exceeds the number of unique patients. After excluding 479 cases of invasive carcinoma with DCIS components, 44 of the remaining 307 pure DCIS cases did not receive the planned RT at our department for variable reasons and were therefore not analyzed. Ultimately, 263 cases met our criteria for analysis (Figure 1). Patient-, tumor-, and treatment-specific characteristics were collected retrospectively from physical patient records and the RT planning system (Varian Eclipse, version 15.6, Varian Medical Systems, Palo Alto, CA, USA).

### 2.2. Patient Population

Prior to RT, all 263 cases were discussed in an interdisciplinary tumor conference and evaluated for possible risk factors that would make recurrence likely. High-risk DCIS was defined as tumor size ≥ 2.5 cm, poor differentiation (G3), age ≤ 50, and a symptomatic initial presentation, such as nipple discharge or a palpable mass. Low-risk DCIS was defined as tumor size < 2 cm, G1/G2, age > 50, and an asymptomatic initial presentation. If a patient did not fully meet all criteria for either category, the case was classified as intermediate risk DCIS. RT was offered to all patients at the University Medical Center Göttingen (UMG). Of the 263 cases, 58 (22.1%) were classified as low risk, 204 (77.6%) as intermediate risk, and 1 (0.4%) as high risk. The definitions of low-, intermediate-, and high-risk disease are based on the best available clinical evidence and the evidence-based recommendations of the Breast Committee of the Arbeitsgemeinschaft Gynäkologische Onkologie (German Gynecological Oncology Group, AGO). Similar approaches to DCIS risk stratification using clinical and pathological factors have been reported by McCormick et al. and Wehner et al. [11,12,13]. With this stratification we tried to reflect everyday routine and clinical practice, where recommendations for or against RT are not always that clear.

In 262 (99.6%) cases, the patients were female, whereas 1 (0.4%) was male. Mean age at initial diagnosis was 59 years (min–max: 36–80). Contralateral DCIS outside the 2008–2020 inclusion period was detected in 4 (1.5%) cases. In 9.9% (*n* = 26) of cases, the abnormal finding was self-detected rather than found through screening. Similar proportions have been reported in the literature; for example, Barnes et al. described symptomatic presentation in approximately 13% of pure DCIS cases [14]. At the time of analysis in September 2024, 34 (14.4%) patients had died.

### 2.3. Tumor and Treatment Characteristics

All patients were diagnosed by biopsy and underwent surgery. In case of close margins (<2 mm), re-resection was performed. All patients were offered adjuvant RT to reduce the risk of recurrence in accordance with current treatment standards. Normofractionated RT was delivered over 5 weeks with doses of 50 or 50.4 Gy in 25 or 28 fractions (2 Gy or 1.8 Gy per fraction). Techniques used were 3D conformal RT (3D-CRT) or, in rare cases because of anatomical special features, Volumetric Modulated Arc Therapy (VMAT).

### 2.4. Radiation Side Effects

Acute toxicities occurring within 90 days after start of radiotherapy were documented according to the Common Terminology Criteria for Adverse Events (CTCAE, version 5.0) [15]. Attending physicians performed physical examinations and assessments at least once per week during therapy and two weeks thereafter at a regular appointment. Whenever side effects had not resolved by that time, patients were monitored closely until sustained clinical improvement. This approach ensured comprehensive appraisal of skin toxicity. The adverse events, which were systematically recorded and classified at the time of reporting, have been archived and can be viewed at any time.

With respect to late toxicity, a minimum follow-up of 5 years was conducted. Late toxicities during this period were documented using the Late Effects Normal Tissue—Subjective, Objective, Management, Analytic (LENT–SOMA) scales. Assessed items comprised fibrosis, hyperpigmentation, telangiectasia, and breast edema. Regular follow-up visits were scheduled on three occasions at typical intervals of 18–20 months after radiotherapy. In case of patient complaints, additional visits were offered immediately, thereby providing high sensitivity for toxicity detection.

Both acute and late toxicities were extracted uniformly by the same investigator. To incorporate all available information, the above-described documentation from the medical records was viewed together with physicians’ letters, which were regularly issued two weeks after completion of RT and whenever relevant toxicities were noted during the five-year aftercare period. Follow-up evaluation was further supplemented by the hospital’s internal documentation systems (ixserv.4, version R20.3, ix.mid software technology, Cologne, Germany) and ONKOSTAR (version 2.11.1.1, IT-Choice Software AG, Karlsruhe, Germany).

### 2.5. Questionnaires on Quality of Life and Decisional Regret

In late 2024 and early 2025, all patients were contacted to obtain consent to complete the European Organisation for Research and Treatment of Cancer Quality of Life Questionnaire (EORTC QLQ-C30) and the Ottawa Decisional Regret Scale (DRS) in German. All surveys are standard evaluation instruments that have been translated into German and previously validated. The QLQC30 has received validation from the EORTC and is accessible in German; the DRS was translated and validated by the authors, led by Rühle et al. [16,17,18]. Using postal addresses and contact details retrieved from the cancer registry and our internal documentation systems, questionnaires as well as a brief patient information and written consent was obtained by the patients. A response rate of 35.4% (93 of 263 questionnaires) was obtained (see Table S4). This single-center study was conducted in accordance with the Declaration of Helsinki and approved by the Ethics Committee of the UMG (protocol code: 2724; approval date: 20 August 2024).

### 2.6. Statistical Analysis

Data analyses were performed using SPSS software (v.26). Associations between clinical features, toxicity items, and responders vs. non-responders to the questionnaires were assessed using the chi-square test or, were appropriate, the chi-square test for trend (also known as Cochran-Armitage test). For clinical outcome, survival times were analyzed as ipsilateral breast recurrence-free survival (IBRFS) and OS. IBRFS and OS were calculated from the end of RT until recurrence or death. IBRFS represented a combined local endpoint of any recurrence (invasive and non-invasive), whereas OS reflected any kind of death. Cumulative recurrence and survival rates were determined using the Kaplan–Meier (KM) method [19]. To test for impact of single variables on recurrence or survival rates, log-rank tests were performed. In addition, patient, tumor, and treatment characteristics were analyzed by univariable Cox regression analyses to determine the respective hazard ratios. Questionnaire data were analyzed using both scale-level and item-level approaches. Associations among the DRS total score, QLQ-C30 scale scores, and late toxicities were examined. In addition, item-level analyses were performed to explore and test hypotheses about certain symptoms without distortion from other items. These analytical approaches were consistent with the recommendations outlined in the EORTC QLQ-C30 and DRS scoring manual [16,17,20,21]. Strength and statistical significance between the aforementioned parameters were assessed using the non-parametric Kendall’s tau-b correlation coefficient.

## 3. Results

### 3.1. Tumor Characteristics

The tumor size was <2 cm in 130 (49.4%) cases and ≥2.5 cm in 67 (25.5%) cases. The tumors were well differentiated (G1) in 51 (19.4%) cases, moderately differentiated (G2) in 98 (37.3%) cases, and poorly differentiated (G3) in 113 (43%) cases; grade was unknown in 1 (0.4%) case. Comedo-type necrosis was present in 43 (16.3%) cases. Surgical margins ≥ 2 mm were achieved in 133 (50.6%) cases, with re-excision performed in 118 (44.9%) to obtain these margins. Overall, 189 (71.9%) cases were estrogen receptor-positive and 136 (51.7%) were progesterone receptor-positive. 97 (51.3%) received hormone therapy, 80 (42.3%) did not, and therapy status was unavailable in 12 (6.3%). Please refer to Table 1 for baseline patient, tumor, and treatment characteristics.

### 3.2. Treatment Outcome and Toxicity

**Figure 2:**
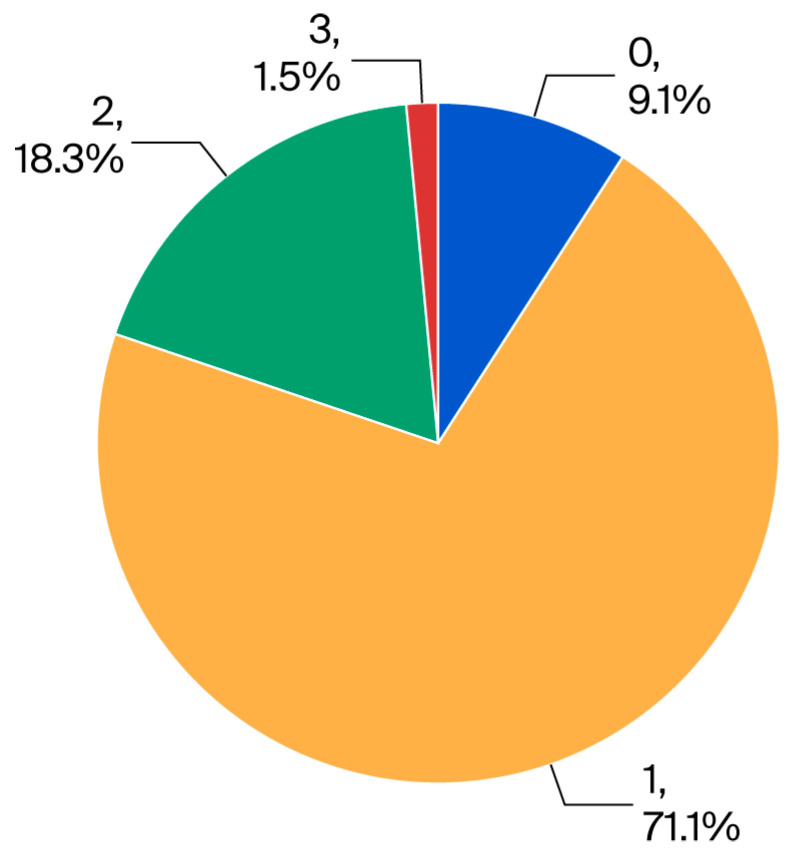
Pie chart of acute cutaneous toxicity grades in patients treated with radiotherapy (*n* = 263).

**Figure 3:**
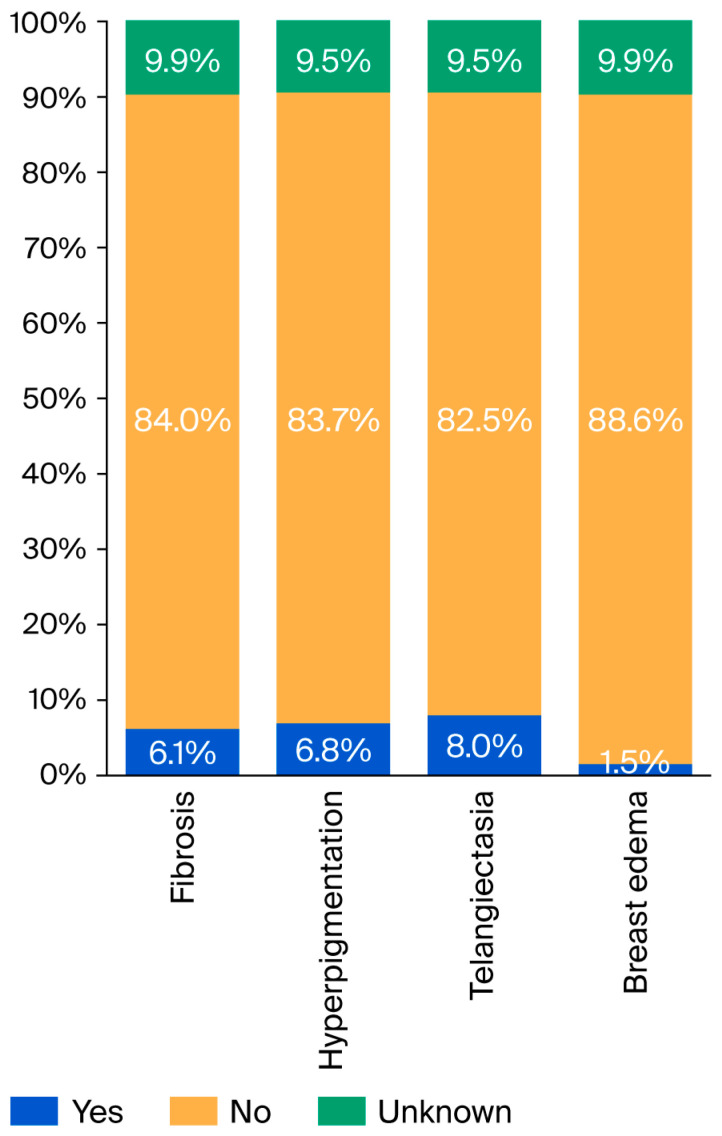
Bar chart of late toxicities after radiotherapy (*n* = 263).

In the entire cohort, median time between surgery and start of radiotherapy was 61 days (25–75% percentile: 48 -77, min–max: 5–401 days). The respective numbers for patients without and with re-excision were 49 (41–61, min–max 5–210) and 74 days (63–87, min–max 26–401). The delay in the re-excision group compared to those with only one surgery was statistically significant (*p* < 0.001, Mann–Whitney U test). RT was discontinued at 46 Gy in one case but included in the analysis; all other patients received the full dose of 50 Gy; a local tumor boost was not administered. Acute skin toxicity was grade 1 in 187 (71.1%) cases, grade 2 in 48 (18.3%) cases, grade 3 in 4 (1.5%) cases, and absent (grade 0) in 24 (9.1%) cases (Figure 2). Chronic toxicities were not mutually exclusive and included fibrosis in 16 (6.1%) cases, hyperpigmentation in 18 (6.8%) cases, telangiectasia in 21 (8%) cases, and edema in 4 (1.5%) cases (Figure 3). As of September 2024, recurrence had occurred in 24 cases, equally split between 12 (50%) invasive cases and 12 (50%) non-invasive cases. The median time from completion of RT to recurrence was 49 months (min–max 10–190).

**Figure 4:**
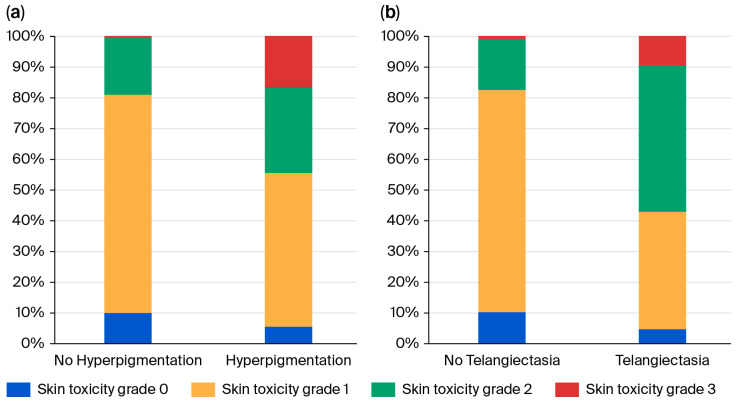
(**a**) Association between acute cutaneous toxicity grading and the occurrence of late hyperpigmentation (*p* = 0.001). (**b**) Association between acute cutaneous toxicity grading and the occurrence of late telangiectasia (*p* = 0.00006). Statistical assessment was performed by chi-square test for trend.

Increasing acute toxicity grades were positively correlated with two items of late toxicities, consistent with consequential late effects, i.e., hyperpigmentation (entire cohort *p* = 0.001 according to chi-square test for trend); intermediate-risk *p* = 0.00005) and telangiectasia (entire cohort *p* = 0.00006; intermediate-risk *p* = 0.000006) (Figure 4).

**Figure 5:**
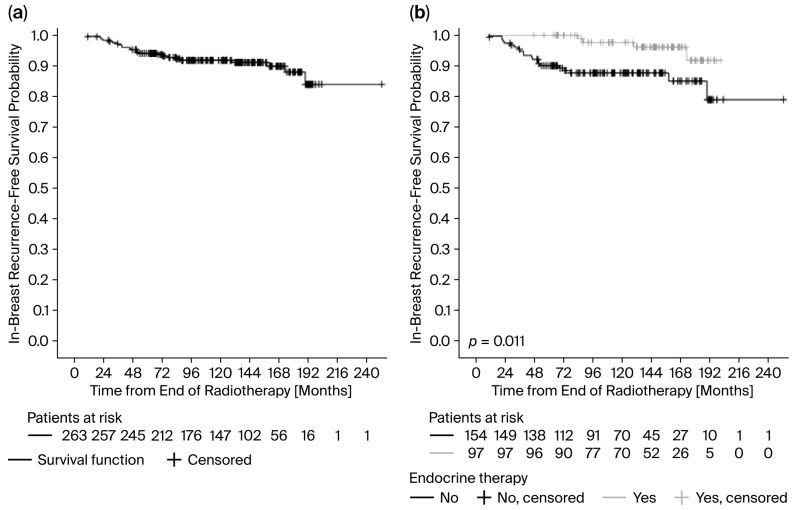
(**a**) Kaplan–Meier estimate of recurrence-free survival for the entire cohort. (**b**) Kaplan–Meier estimate of recurrence-free survival stratified by endocrine therapy. Data on adjuvant therapy were unavailable for twelve cases with positive estrogen and/or progesterone receptor status; therefore, panel b includes 251 instead of 263 cases.

Median follow-up time was 129 months (25–75% percentile: 83–163 months, min–max 10–253). Out of 263 patients, 24 local recurrence events were registered. Un-stratified global IBRFS rate was 92% and 88% at 10 and 15 years, respectively (Figure 5a). Analyses were conducted to assess potentially predictive factors for this metric. When evaluating initial tumor size over all five categories, there was no impact on IBRFS (*p* = 0.807, log rank test for trend). Moreover, when comparing tumors ≥ 5 cm with all smaller categories combined, again no statistically significant difference in recurrence risk was observed (*p* = 0.158). Tumor grade considered as three ordinally ordered categories (*p* = 0.388) and acute skin toxicity (four grades 0–3, *p* = 0.734) were not related to IBRFS. Variables in binary fashion were evaluated by univariable Cox regression analyses with respect to IBRFS (Table 2). Estrogen receptor-positive cases showed a trend toward a lower risk of ipsilateral recurrence (*p* = 0.096). Receipt of endocrine therapy was linked to reduced recurrence risk (*p* = 0.017, see also Figure 5). The occurrence of hyperpigmentation, a late radiotherapy sequela, was linked to an increased risk for IBRFS events, with a hazard ratio of 4.38 (95% confidence interval 1.61–11.90, *p* = 0.004). However, only 18 patients were affected by this long-term side effect (Table 2). Ten-year IBRFS was 93.6% and 72.2% when hyperpigmentation was absent and present, respectively. In addition, telangiectasia was more frequent on the left than on the right side (risk ratio 2.70, 95% confidence interval 1.02–7.14, *p* = 0.034 chi-square test). This impact of sidedness was retained in a binary logistic regression model when adjusting for acute skin toxicity ≥ grade 2, which identified as strong determinant of telangiectasia, Figure 4b). As we here report exploratory analyses, they are not adjusted for multiplicity testing. Given the low numbers of recurrence events, multivariable models were not employed.

Regarding overall survival (OS), none of the tested variables reached an association at *p* < 0.05. A statistical trend was observed only for patient age at DCIS diagnosis, with an increased hazard ratio for patients older than 50 years. Notably, the occurrence of ipsilateral tumor recurrence did not confer a relevant risk with respect to OS (Table 3).

### 3.3. QoL and DR

**Figure 6:**
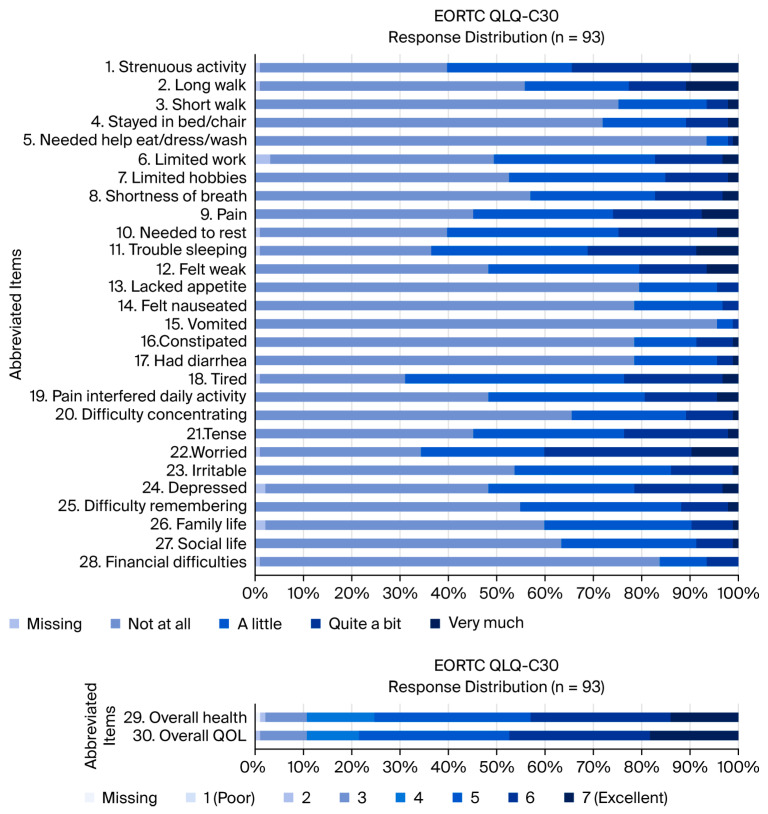
Response distribution for all items of the EORTC QLQ-C30 questionnaire (*n* = 93).

**Figure 7:**
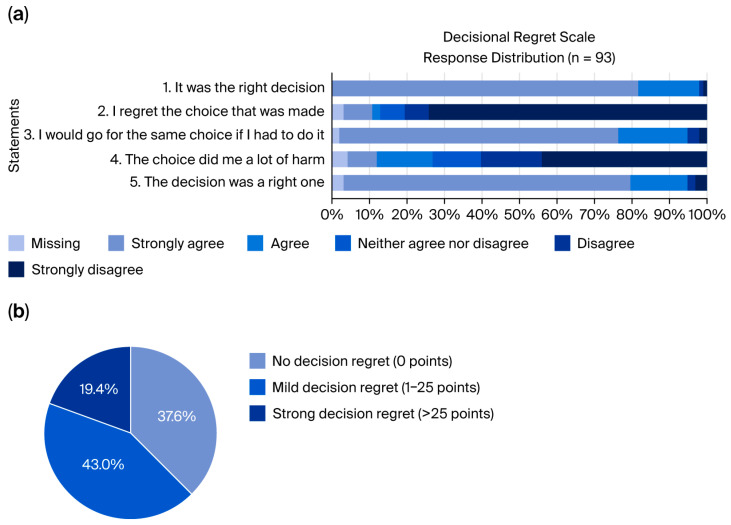
(**a**) Response distribution for the five items of the Decisional Regret Scale (*n* = 93). (**b**) Distribution of overall decisional regret categories (no, mild, and strong regret) based on total scale scores.

The EORTC QLQ-C30 and DRS were scored according to their respective user manuals (Table 4). Acceptability was based on response rate, number of missing items, completion time and items perceived as confusing or difficult to answer. An overview of item response distribution is shown in Figure 6. Completion time for both questionnaires when administered on the phone ranged from 5 to 15 min. A total of 93 (35.1%) EORTC and DRS questionnaires were obtained. Patient, tumor, and treatment characteristics were balanced between responders and non-responders for most variables (see Supplementary Material Table S4). Minor deviations were seen with DCIS laterality (reduced questionnaire recall when the tumor was on the left side, *p* = 0.042) and an increased incidence of non-responders in cases of acute cutaneous toxicity ≥ grade 2 (*p* = 0.039). Among the 93 patients who responded to the questionnaire, 22 were classified as low-risk, 70 as intermediate-risk, and none as high-risk. This distribution did not differ from that of the total cohort (*p* = 0.35 according to the chi-square test). Of otherwise completed questionnaires, 15 QLQ-C30 items and 12 DRS items were left unanswered (Figure 1). In addition, overall DR was categorized based on total DRS scores as no regret (score = 0), mild regret (score 1–25), and strong regret (score > 25). Response frequencies and categorization are shown in Figure 7. Only 3 of the patients who generally regretted their treatment had a recurrence; none of them had invasive carcinoma. However, a causal relationship cannot be established here because the DRS does not allow us to determine why a treatment is regretted, only whether it is regretted.

Within the QLQ-C30, a clear correlation structure was observed (see Supplementary Material, Table S1, with sheets for raw and multiplicity-adjusted *p*-values). The five functional scales (Physical, Role, Emotional, Cognitive, and Social Functioning) were strongly positively correlated with each other (all p_adjusted_ < 0.01 after Bonferroni correction for multiple testing). Within the symptom scales, several strong positive intercorrelations were observed, the strongest being between fatigue and pain (Kendall’s tau-b coefficient = 0.623, p_adjusted_ = 0.0001). Moreover, many symptom scales—particularly fatigue, pain, dyspnea, and insomnia—exhibited strong and clinically plausible correlations with all or most functional scores. In contrast, this was not the case for diarrhea or constipation. Financial difficulties, in turn, were associated with social functioning (p_adjusted_ < 0.05) and, to a lesser extent, with emotional functioning (p_adjusted_ < 0.02), but not with physical, role, or cognitive functioning. For interpretation of the correlation directions, it should be noted that higher scores on the functional scales indicate better functioning, whereas higher scores on the symptom scales indicate greater symptom burden. The Global Health Status/QoL score was positively correlated with the functional scales and with most symptom scales. Interestingly, DRS was neither related to the overall QoL score nor to any of its functioning or symptom subscales.

Correlation analyses of overall QoL with late toxicities did not reach statistical significance for any of the four investigated single late-toxicity items. When the occurrence of any late toxicity was considered, regardless of its type or intensity, a weak negative correlation with overall quality of life was observed (Kendall’s tau-b coefficient of −0.193, *p* = 0.037). This relationship was deemed exploratory (i.e., hypothesis-generating and not -proving) without adjustment for multiplicity testing. With respect to the DRS score, no correlation with late toxicities was noted. When these analyses were restricted to the subset of patients with an intermediate DCIS risk profile, similar relations were detected. In this case, in addition to the combined toxicity variable, the single items “hyperpigmentation” and “breast edema” also reached unadjusted *p*-values < 0.05 for correlations with the global quality-of-life score (see Supplementary Material, Tables S2 and S3).

## 4. Discussion

In this study, we observed excellent progression-free and overall survival in 263 cases with DCIS after RT. Patient-reported outcomes were also reassuring among 93 respondents (35.4%), with long-term QoL impairment generally low, and strong DR uncommon, regardless of recurrence status or risk category. Although predicting which DCIS lesions may progress to invasive disease remains challenging, and despite ongoing concerns about overtreatment, these results support offering RT to patients with low- or intermediate-risk DCIS when the goal is to minimize long-term recurrence risk.

### 4.1. Characteristics

The patient, tumor, and treatment characteristics observed in our cohort are broadly in agreement with those reported in the literature. Specifically, the population was almost exclusively female with a median age of 59 years, and most cases were detected at screening. Tumor size and grading distributions were comparable to those reported in randomized trials and population-based studies, although larger lesions were slightly less frequent in our cohort. Grade I DCIS represented the least common histological subtype. Treatment patterns, including the use of adjuvant therapy and endocrine therapy, were also in line with routine clinical practice. These similarities support the compatibility of our cohort with published data and help place our recurrence analyses into the context of existing evidence [2,22,23]. Furthermore, symptomatic presentation, size, margin status, nuclear grade or presence of Comedo-type necrosis were not associated with recurrence. This may, at least in part, be explained by the uniform use of adjuvant RT in our cohort, which could have reduced the prognostic impact of these variables [24].

### 4.2. Treatment Outcomes and Toxicity

Freedom from ipsilateral breast recurrence reached 88% at 15 years in our cohort, using a composite endpoint of invasive and non-invasive events. Comparable rates have been reported by Donker et al., who observed a 15-year ipsilateral recurrence-free rate of 82% [25]. Direct comparison is limited by a missing control group and unequal follow-up durations across studies.

Overall, comparisons with the literature are further constrained by the small sample size of this study, which precluded the detection of differences between invasive and non-invasive recurrences. Another limitation is particularly relevant for low- and intermediate-risk patients, in whom recurrence events may take many years to develop. Indeed, Sanders et al. report that the natural history of low-grade DCIS may extend over several decades, potentially exceeding four decades, raising the question of whether recurrence would occur within a patient’s remaining life expectancy [26].

The use of endocrine therapy significantly decreased the ipsilateral recurrence rate. This finding is consistent with the existing literature, supporting the addition of endocrine therapy to surgery and RT to further reduce the risk of breast events, at least in cases with high-risk factors, as previously defined [27]. Endocrine therapy has also been shown to reduce the risk of contralateral breast cancer; notably, only one patient in our cohort developed contralateral DCIS despite receiving endocrine therapy for more than one year before discontinuation due to side effects. However, the low event rate and absence of assessment of contralateral invasive events limit interpretation [27,28].

The relevance of hyperpigmentation as a potential surrogate marker for IBRFS elicited in this exploratory analysis remains elusive despite a relatively high hazard ratio. To the best of our knowledge, as this specific association has not been addressed in literature thus far, it might warrant further investigation in future studies. In contrast, the higher incidence of telangiectasia on the left side might be a consequence of differential radiation beam arrangements. The necessity to spare the heart as much as possible may result in increased skin exposure, leading to a higher rate of telangiectasia in conjunction with individual vascular responses. However, this finding should be interpreted with caution, as the association was weak. Comparable relations between sidedness and fibrosis or hyperpigmentation were not observed. RT-related acute toxicities were predominantly mild: 71.1% of cases exhibited only grade 1 acute cutaneous reactions, and only two cases required premature discontinuation of RT due to acute related complications. Late toxicities occurred at acceptable rates, with fibrosis observed in 6% of cases, hyperpigmentation in 6.1%, and telangiectasia in 8%. These findings fall within the range of previously published data. For example, Bourgier et al. reported grade 2–3 breast subcutaneous fibrosis in 2.8% of patients, alongside higher rates of lower-grade fibrosis [29]. Importantly, we observed a significant association between acute and chronic skin effects. Higher grades of acute cutaneous toxicity were associated with increased rates of late hyperpigmentation and telangiectasia, with *p*-values of 0.001 and 0.00006, respectively. This finding is consistent with the concept of consequential late effects, as described by Bentzen, whereby severe acute epithelial damage predisposes to persistent microvascular damage and subsequent late skin changes [30]. In the context of DCIS patients, this association is particularly relevant, as patients generally have excellent long-term survival, rendering late treatment-related toxicity a key outcome of interest. Notably, late toxicities showed no consistent association with overall QoL or DRS scores. Only a weak nominal correlation with quality of life was observed when any toxicity was present, and similar weak associations, particularly for hyperpigmentation and breast edema, were seen in patients with an intermediate risk profile.

### 4.3. QoL and DR

A major strength of this study is the relatively high response rate of 35.4% compared with other investigations of survivorship after DCIS treatment, such as in the study by Sinclair et al., which reported a response rate of 28.7%. Responders and non-responders did not differ with respect to DCIS risk distribution. As 92 respondents belonged to the low- or intermediate-risk group, they represent the very group being studied in de-escalation and active surveillance trials.

Sinclair et al. further showed that RT was associated with a lower fear of recurrence, emphasizing that psychosocial factors are important in decision-making. These data highlight the value of shared clinician–patient discussions that consider both recurrence risk and personal reference when deciding whether to include RT [31]. In our cohort, the median global health status and quality-of-life-score was 71.1, while median functional scale scores ranged from 71.9 to 83.9, indicating generally preserved QoL. DR, defined as a preference for an alternative treatment outcome, represents another relevant consideration when comparing RT with omission strategies [18]. Given the risk profile of the cohort, the mean DRS score was 14, with strong regret reported by only 19.4% of respondents, suggesting that most patients did not experience substantial regret regarding their treatment choice. Moreover, the DRS showed only weak correlations with the other parameters. Slight negative correlations were observed with the functional scales, except for Cognitive Functioning (*p* > 0.05), whereas weak positive correlations were found with Fatigue and Dyspnea. According to the EORTC scoring manual, the Global Health Status/QoL scale (Q29, Q30) should be used as the overall summary measure. No meaningful association between QoL or the QLQ-C30 sub-scales with DRS was identified. Consequently, there are no strong grounds to assume that variations in functional status or symptom burden substantially influenced patients’ decisions, as reflected by the DRS.

Nonetheless, considering the overall low response rate—albeit relatively high compared to other studies (Sinclair et al.)—there is an increased possibility that stratifying factors may be less well balanced between responders and non-responders of the questionnaires. Careful assessment of this issue revealed laterality and acute cutaneous toxicity ≥ grade 2 as such potentially confounding factors (Supplementary Material Table S4). Given the rather weak associations, with *p*-values only slightly below 0.05 and no longer statistically insignificant after Bonferroni correction, it is not unlikely that these two imbalances occurred by chance. However, an influence on the here-reported results cannot be completely ruled out. While a potential impact of laterality on questionnaire response remains elusive, it is conceivable that incurred toxicity may affect patients’ willingness to respond. One might expect that late toxicity could provoke unwillingness of patients to participate in measures reminding them of radiotherapy and its related side effects. However, it was not late, but acute toxicity which was associated with the recall rate. Considering the minimum interval of 59 months (median 142) between end of radiotherapy and questionnaire delivery, it appears questionable whether patients truly remembered the acute toxicity and whether this influenced their decision to respond. Sensitivity analyses were conducted for the combined score of overall health and quality of life (item #29 and #30 of the QLQ-C30) and for the combined final DRS score in the subgroup of patients who responded to the questionnaire. No significant association were observed with laterality and acute toxicity ≥ grade 2 (all four *p*-values ≥ 0.2 as assessed by Mann–Whitney U test). Furthermore, these two factors showed no significant impact on IBRFS (Table 2) or OS (Table 3).

## 5. Limitations and Conclusions

In summary, the main limitations of this study include issues such as missing or incomplete documentation, the biological and clinical heterogenicity of DCIS, and variability in treatment regimens, including differences in endocrine therapy type and duration, which may have diluted the isolated effect of RT. An inherent limitation of this study is the lack of a group of patients with comparable characteristics (in particular, intermediate risk) who had an optional indication for radiotherapy but declined treatment. As such patients typically present to radiotherapy departments to a much lesser extent, an allocation bias must be considered. To circumvent this, a broader spectrum of clinicians involved in the treatment of patients with intermediate-risk DCIS should be part of future study designs. Although we are currently not able to address this issue adequately, our data may provide a rational for stimulating further research in this field, including multi-arm clinical trials.

The duration of the follow-up (median 129, min–max 10–253 months) in our study might be regarded as relatively short with respect to the outcome “recurrence”, as this endpoint may also manifest beyond a decade due to the biological characteristics of DCIS. However, given the median time between completion of radiotherapy and recurrence of 49 months (min–max 10–190 months), this issue seems to be fairly well covered by the follow-up period in our sample. Moreover, this time frame seems adequate for assessing treatment-related side effects and quality of life (QoL) outcomes.

Another significant critique to consider when interpreting the data is that some surveys were completed over a decade after the conclusion of radiation therapy, perhaps resulting in memory distortion, especially concerning the acute side effects observed. Nonetheless, these were not evaluated using the questionnaires but were extracted from the accessible medical data. The questionnaires explicitly inquired about participants’ current well-being, any residual side effects, ongoing limitations in daily activities, and any regrets concerning the therapy based on present knowledge, thereby mitigating memory bias to the greatest extent possible. Despite these limitations, we observed excellent rates of recurrence-free survival and acceptable rates of late toxicity. Based on these findings, RT should be regarded as a valuable treatment option for the subgroup of interest while underscoring the importance of individualized, case-by-case decision-making. To our knowledge, there remains a paucity of studies addressing QoL and DR following RT for DCIS, and the present study contributes meaningful data to this underrepresented area of DCIS survivorship research.

## Figures and Tables

**Table 1:** Baseline patient, tumor, and treatment characteristics of the study cohort. Data refer to numbers (percentages) of parameters if not otherwise specified.

Entire Study Cohort	*n* = 263 (100%)
***Patient characteristics***	
Age at initial diagnosis in years—median (min–max)	59 (36–80)
Female	262 (99.6)
Male	1 (0.4)
***Mode of detection***	
Screen-detected	233 (88.6)
Clinical	26 (9.9)
Unknown	4 (1.5)
***Tumor characteristics***	
Contralateral DCIS	4 (1.5)
*Laterality*	
Right breast	122 (46.4)
Left breast	141 (53.6)
*Tumor size*	
<1 cm	56 (21.3)
≥1 cm to <2 cm	74 (28.1)
≥2 cm to <3 cm	42 (16)
≥3 cm to <4 cm	26 (9.9)
≥4 cm	24 (9.1)
Unknown	41 (15.6)
*Grading*	
Grade I	51 (19.4)
Grade II	98 (37.3)
Grade III	113 (43)
Unknown	1 (0.4)
Comedo-type necrosis	43 (16.3)
Surgical margins ≥ 2 mm	133 (50.6)
Re-excision	118 (44.9)
*Hormone receptor status*	
Estrogen-receptor-positive	189 (71.9)
Progesterone-receptor-positive	136 (51.7)
HER2-positive	37 (14.1)
***Treatment characteristics***	
Days from surgery to the initiation of radiotherapy—median (min–max)	61 (5–401)
Endocrine therapy when ER-positive	97 (36.9)

ER: estrogen receptor.

**Table 2:** Influence of independent variables on IBRFS. Univariable Cox proportional hazards models were employed. Hazard ratios are presented with 95% confidence intervals (CI). Respective patient numbers are denoted in brackets.

Variable	Hazard Ratio (95% CI)	*p*-Value
Patient age at DCIS diagnosis: >50 (229) vs. ≤50 y (34)	0.45 (0.18–1.13)	0.090
Symptomatic disease: yes (26) vs. no (233)	0.85 (0.20–3.62)	0.825
Tumor size: ≥2.5 (67) vs. <2.5 cm (155)	1.12 (0.46–2.74)	0.807
Laterality: Left (141) vs. right (122)	0.91 (0.41–2.02)	0.808
Grading: G3 (113) vs. G1–2 (149)	1.07 (0.48–2.39)	0.872
Risk profile: intermediate (204) vs. low (58)	1.39 (0.48–4.07)	0.547
Comedo-type necrosis: yes (43) vs. no (220)	0.22 (0.03–1.66)	0.144
ER-positive (189) vs. -negative (62)	0.48 (0.21–1.14)	0.096
PR-positive (136) vs. -negative (112)	0.59 (0.25–1.38)	0.220
HER2 receptor: positive (37) vs. negative (77)	3.63 (1.06–12.4)	0.040
Endocrine therapy: yes (97) vs. no (154)	0.27 (0.09–0.79)	0.017
Acute skin toxicity: grades 2–3 (52) vs. 0–1 (211)	1.33 (0.53–3.34)	0.551
Late Toxicities		
Fibrosis, yes (16) vs. no (221)	0.55 (0.07–4.11)	0.559
Hyperpigmentation, yes (18) vs. no (220)	4.38 (1.61–11.9)	0.004
Telangiectasia, yes (21) vs. no (217)	0.91 (0.21–3.90)	0.900

ER: Estrogen receptor, PR: progesterone receptor.

**Table 3:** Impact of independent variables on OS. Univariable Cox proportional hazards models were applied. Hazard ratios are presented with 95% confidence intervals (CI). Respective patient numbers are shown in brackets.

Variable	Hazard Ratio (95% CI)	*p*-Value
Patient age at DCIS diagnosis: >50 (229) vs. ≤50 y (34)	5.39 (0.74–39.4)	0.097
Symptomatic disease: yes (26) vs. no (233)	1.51 (0.58–3.90)	0.395
Tumor size: ≥2.5 (67) vs. <2.5 cm (155)	0.88 (0.37–2.10)	0.778
Laterality: Left (141) vs. right (122)	1.36 (0.68–2.69)	0.382
Grading: G3 (113) vs. G1–2 (149)	0.71 (0.35–1.43)	0.331
Risk profile: intermediate (204) vs. low (58)	0.68 (0.32–1.42)	0.299
Comedo-type necrosis: yes (43) vs. no (220)	1.53 (0.69–3.39)	0.291
ER-positive (189) vs. -negative (62)	0.89 (0.42–1.93)	0.775
PR-positive (136) vs. -negative (112)	1.19 (0.60–2.38)	0.617
HER2 receptor: positive (37) vs. negative (77)	1.00 (0.41–2.46)	0.997
Endocrine therapy: yes (97) vs. no (154)	1.06 (0.54–2.11)	0.862
Acute skin toxicity: grades 2–3 (52) vs. 0–1 (211)	1.54 (0.74–3.22)	0.252
Late toxicities		
Fibrosis yes (16) vs. no (221)	1.19 (0.36–3.96)	0.782
Hyperpigmentation yes (18) vs. no (220)	0.58 (0.08–4.22)	0.587
Telangiectasia yes (21) vs. no (217)	1.51 (0.58–3.96)	0.398
Ipsilateral relapse: yes (12) vs. no (251)	2.05 (0.62–6.73)	0.237

ER: Estrogen receptor, PR: progesterone receptor.

**Table 4:** Summary of EORTC QLQ-C30 scale scores and Decisional Regret Scale totals.

Scale	Median	Q1–Q3	Min–Max	*n*	Item Numbers
**EORTC QLQ-C30**					
***Functional scales***					
Physical functioning	87	73–100	13–100	93	1–5
Role functioning	83	67–100	0–100	93	6, 7
Emotional functioning	75	50–100	25–100	93	21–24
Cognitive functioning	100	67–100	0–100	93	20, 25
Social functioning	100	67–100	33–100	93	26, 27
***Symptom scales***					
Fatigue	22	11–44	0–89	93	10, 12, 18
Nausea and vomiting	0	0–0	0–67	93	14, 15
Pain	17	0–50	0–100	93	9, 19
Dyspnea	0	0–33	0–100	93	8
Insomnia	33	0–67	0–100	92	11
Appetite loss	0	0–0	0–67	93	13
Constipation	0	0–0	0–100	93	16
Diarrhea	0	0–0	0–100	93	17
Financial difficulties	0	0–0	0–67	92	28
***Global health status/QoL***	67	58–83	17–100	93	29, 30
**Decision Regret Scale**					
Final Score	10	0–25	0–85	93	1–5

Values are presented as mean ± standard deviation and median (min–max). Higher scores on functional scales and global health/QoL indicate better functioning, whereas higher scores on symptom scales and decisional regret indicate greater symptom burden or regret.

## Data Availability

The original contributions presented in this study are included in the article and Supplementary Material. Further inquiries may be directed to the corresponding author.

## Supplementary Materials

The following supporting information can be downloaded at: https://www.mdpi.com/article/10.3390/cancers18121946/s1, Table S1: Kendall’s tau-b correlation of functional, symptom and Global Health Status/QoL-scales; Table S2: Kendall’s tau-b correlation of late toxicities and QoL/DRS; Table S3: Kendall’s tau-b correlation of late toxicities and QoL/DRS for patients with intermediate grade DCIS; Table S4: Questionnaire recall rates stratified by patient, tumor and treatment characteristics.

## Abbreviations

The following abbreviations are used in this manuscript:

DCIS	Ductal Carcinoma In Situ
BCS	Breast Conserving Surgery
RT	Radiotherapy
OS	Overall Survival
SDM	Shared Decision Making
QoL	Quality of Life
DR	Decisional Regret
3D-CRT	3D-Conformal Radiotherapy
VMAT	Volumetric Modulated Arc Therapy
DRS	Decisional Regret Scale
CTCAE	Common Toxicity Criteria of Adverse Events
LENT-SOMA	Late Effects Normal Tissue—Subjective, Objective, Management, Analytic
EORTC QLQ-C30	European Organisation for Research and Treatment of Cancer Quality of Life Questionnaire
IBRFS	Ipsilateral Breast Recurrence-Free Survival
KM	Kaplan–Meier
CI	Confidence Interval
ER	Estrogen Receptor
PR	Progesterone Receptor
