# Supplementary material for: Radiotherapy for Ductal Carcinoma In Situ: Toxicity, Quality of Life, and Decisional Regret at a Tertiary Cancer Center
Source: Cancers (Basel). 2026 Jun 16;18(12):1946. doi: 10.3390/cancers18121946 (PMC13297482; doi:10.3390/cancers18121946)
Supplement: Supplementary file 1 [file cancers-18-01946-s001.zip › Table S4.pdf]

	Responders (%)	Non-Responders (%)	*p*-Value
**Entire Study Cohort**	**93 (35)**	**170 (65)**	---
Age at initial diagnosis			
≤ 50 years	13 (38)	21 (62)	0.707
> 50 years	80 (35)	149 (65)	
Mode of detection			
Screen-detected	83 (36)	150 (64)	0.904
Self-detected	9 (35)	17 (65)	
Unknown	1 (25)	3 (75)	
Tumor characteristics			
Laterality			
Right breast	51 (42)	71 (58)	0.042
Left breast	42 (30)	99 (70)	
Tumor size			
< 2.5 cm	57 (37)	98 (63)	0.728
≥ 2.5 cm	21 (31)	46 (69)	
Unknown	15 (37)	26 (63)	
Grading[1]			
Grade I/II	52 (35)	97 (65)	0.817
Grade III	41 (36)	72 (64)	
Comedo-type necrosis			
No	82 (37)	138 (63)	0.142
Yes	11 (26)	32 (74)	
Surgical margins			
< 2mm	46 (35)	87 (65)	0.790
≥ 2 mm	47 (36)	83 (64)	
Re-excision			
No	55 (38)	90 (62)	0.334
Yes	38 (32)	80 (68)	
Recurrence[2]			
No	82 (34)	157 (66)	0.260
Yes	11 (46)	13 (54)	
Treatment characteristics			
Endocrine therapy[3]			
No	61 (40)	93 (60)	0.164
Yes	30 (31)	67 (69)	
Acute skin toxicity			
Grade 0-1	81 (38)	130 (62)	0.039
Grade 2-3	12 (23)	40 (77)	
Late toxicity[4]			
No	72 (38)	118 (62)	0.881
Any	18 (37)	31 (63)	

ER: estrogen receptor, PR: progesterone receptor. [1]Grading missing in one patient.[2]Recurrence was defined as combined numbers of ipsilateral invasive and non-invasive relapses. [3]In 12 cases, there was indication for endocrine therapy of which information on application was not at hand. [4]Late toxicity here represents the combined occurrence of fibrosis, hyperpigmentation, teleangiectasia, and breast edema; information hereon is missing for 24 patients.
